# Synthesis of Polyamide-Based Microcapsules via Interfacial Polymerization: Effect of Key Process Parameters

**DOI:** 10.3390/ma14195895

**Published:** 2021-10-08

**Authors:** Angeliki D. Mytara, Konstantina Chronaki, Vasilis Nikitakos, Constantine D. Papaspyrides, Konstantinos Beltsios, Stamatina Vouyiouka

**Affiliations:** 1 Laboratory of Polymer Technology, School of Chemical Engineering, Zographou Campus, National Technical University of Athens, 15780 Athens, Greece; amytara@mail.ntua.gr (A.D.M.); kchronaki@mail.ntua.gr (K.C.); ch14529@mail.ntua.gr (V.N.); kp@cs.ntua.gr (C.D.P.); 2 Department of Materials Science and Engineering, School of Chemical Engineering, Zographou Campus, National Technical University of Athens, 15780 Athens, Greece

**Keywords:** polyamide microcapsules, polyamide 210, interfacial polymerization, encapsulation, microcapsule morphology

## Abstract

Polyamide microcapsules have gathered significant research interest during the past years due to their good barrier properties; however, the potential of their application is limited due to the fragility of the polymeric membrane. Fully aliphatic polyamide microcapsules (PA MCs) were herein prepared from ethylene diamine and sebacoyl chloride via interfacial polymerization, and the effect of key encapsulation parameters, i.e., monomers ratio, core solvent, stirring rate and time during the polymerization step, were examined concerning attainable process yield and microcapsule properties (shell molecular weight and thermal properties, MC size and morphology). The process yield was found to be mainly influenced by the nature of the organic solvent, which was correlated to the diffusion potential of the diamine from the aqueous phase to the organic core through the polyamide membrane. Thus, spherical microcapsules with a size between 14 and 90 μm and a yield of 33% were prepared by using toluene as core solvent. Milder stirring during the polymerization step led to an improved microcapsule morphology; yet, the substantial improvement of mechanical properties remains a challenge.

## 1. Introduction

Encapsulation systems are being constantly developed for three main purposes pertaining to the active compound: (i) shielding of the active compound against environmental conditions such as pH, radiation, temperature and other chemicals, (ii) changing of its physicochemical properties and (iii) more facile and better controlled transfer of the compound to the target [1,2,3,4,5,6]. In particular, microcapsule-based encapsulation systems (MCs) consist of the core material and the surrounded shell, with sizes ranging from 1–1000 μm [7,8,9,10].

The main chemical methods for the synthesis of polymeric microcapsules involve (i) in situ polymerization, (ii) interfacial techniques, (iii) self-assembly methods and (iv) layer-by-layer techniques [2]. In situ polymerization is regarded as a most important and industrially preferred technique for the preparation of core–shell MCs, especially for urea–formaldehyde and melamine–formaldehyde microcapsules for self-healing applications [11,12,13,14,15,16]. In such processes, a solution of the monomeric or oligomeric wall material is added to the core solvent, the latter being dispersed to the desired size. Similarly, with in situ polymerization, interfacial polymerization is a simple technique in which monomers of high reactivity are dissolved into two non-mixing liquids. The reaction takes place on the interphase of the liquids and the polymeric shell is formed, enclosing the core solvent. Through interfacial techniques, thin shell MCs are generated which permit the transfer of a large amount of core materials with a smaller amount of shell materials. Interfacial methods, are the dominant technique for the preparation of polyamide microcapsules, as will be further explained, but are also applied for polyurethanes, polyureas, and polyesters [2,17]. For the fabrication of more complex microcapsules, self-assembly is a procedure in which different structures are generated from building units which could be either molecules or microparticles. These rearrange spontaneously to form some structural patterns via different interactions including hydrogen bonds, van der Waals forces, electrostatic forces, and *p*–*p* interactions without any outside assistance. Therefore, the self-assembly is controlled entirely by the material properties inherent to the building units. This technique has been proved successful especially in encapsulation of phase change materials [18,19]. Finally, layer-by-layer (LBL) methods have gathered recently significant interest in the biomedical field. The LBL method is useful in the control of shell features such as permeability, morphology, biocompatibility and mechanical stability. This method consists of the assembly of multilayer films onto colloidal particles, followed by selective core removal. The assembly is facilitated by electrostatic interaction, hydrogen bonding, covalent bonding, base–pair interactions, guest–host interactions as well as van der Waals interactions between the different layers [2,20,21].

Amongst polymeric shells for microencapsulation, polyamides have gathered research interest during the past years. In comparison to polyesters, polyamide membranes are more resistant to a variety of common solvents and have lower oxygen permeability [22,23]. Furthermore, polyamides are known to have excellent thermal and mechanical properties (melting points between 170–280 °C, good dimensional stability); however, a common problem impeding the use of polyamide microcapsules is associated with shell integrity. According to Chen et al., a main drawback of polyamide and polyurethane microcapsules originates from their thin wall and their weak mechanical properties [24].

In the literature, polyamide microcapsules (PA MCs) are produced via interfacial polymerization, based on the Schotten–Baumann reaction between a polyfunctional amine and an acyl chloride [2,6,25]. Encapsulation via interfacial polymerization takes place in two steps, with the encapsulation of oil-soluble substances the most studied [26,27,28,29,30,31,32,33]. First, the amine and the chloride are dissolved in two immiscible solvents. Most often, organic droplets containing the chloride and the active substance are dispersed in a continuous aqueous phase containing the amine with the help of a dispersion agent/emulsifier. Then, the acyl chloride and the amine molecules diffuse and react (polymerization step). There are also a number of works on the encapsulation of water-soluble substances [34,35,36,37]: the emulsification step involves the dispersion of the aqueous phase containing the amine and the active compound in a continuous organic phase, containing an emulsifier and the chloride. The reaction again takes place on or near the interphase, forming the polyamide membrane which covers the aqueous droplet.

Significant research has been carried out on polyamide microcapsules starting from a range of monomers (aliphatic or aromatic, bifunctional or polyfunctional, etc.) [24,27,31,33,36,38,39,40,41,42,43,44]. The effect of process variables such as ratio of monomers, type and concentration of the emulsifier, emulsification method, and time on the final ΡA MCs properties, especially the size, shell integrity, permeability along with release profiles and mechanisms, has been investigated. However, both the mechanical stability and the release properties through the shell of the MCs, as well as the success of MCs synthesis, depend on the inherent nature of the monomers [2,6,7,40]. Aliphatic chlorides such as sebacoyl and succinyl chloride [6,31,38] have lower reactivity than aromatic ones such as terephthaloyl chloride [6,28,41,45]; however, the former tend to form more favorable conformations to the reactions with diamine due to their flexible aliphatic chain [6]. Furthermore, aliphatic chlorides are more susceptible to hydrolysis, but form less porous and smoother polyamide shells [27,38,42]. On the other hand, aliphatic amines, such as ethylene diamine and 1,8 diamino-octane, are preferred due to their reactivity and hydrophilic nature [6,36,38,44]. The tendency of the amine component to diffuse towards the organic phase is the rate-determining step of interfacial polymerization.

Although aliphatic polyamides have been extensively studied as polymeric materials in the literature, there are very few studies concerning aliphatic polyamide microcapsules [31,34,38,46]. In this context, the present work aims to examine and improve the synthesis of polyamide 210 microcapsules (ΡA 210 MCs) from sebacoyl chloride (SC) and ethylene diamine (EDA). Sebacoyl chloride is less sensitive to hydrolysis, along with terephthaloyl chloride, and has the potential to be synthesized from bio renewable resources, while ethylenediamine has high reactivity. A series of key interfacial polymerization parameters are examined as to process yield, MC morphology, molecular weight and thermal properties of the polyamide membrane. Additionally, in terms of receiving further information about the polyamide shell, the use of two methods is herein proposed: (i) a control experiment and (ii) model (i.e., plane) membranes. In the control experiment, the experimental process is followed in the same way as for the production of MCs without the presence of an emulsifier. In other words, an “unstable” dispersion of the organic droplets containing the chloride, is created in the aqueous medium, followed by the addition of the amine. This method permits the synthesis of a polyamide “capsule-like” membrane in conditions close to the ones applied for the preparation of MCs. The synthesis of plane polyamide membranes, on the other hand, is a method which further simplifies the interfacial polymerization system by including only the two solutions needed for the polycondensation. This method has been previously reported in the literature for investigation of the synthesis of polyamide microcapsules and the extraction of information as regards the polyamide shell in the case of difficulties stemming from the limited quantity and/or fragility of microcapsules [29,31,36,40,44].

## 2. Materials and Methods

### 2.1. Materials 

Sebacoyl chloride (SC) by Alfa Aesar and ethylene diamine (EDA) by Sigma Aldrich were used as monomers for interfacial polymerization. Dodecane (99%, anhydrous) by Sigma Aldrich, hexane (99%) by Merck and toluene (99.8%) by Fischer Chemicals were used as solvents for SC and water for EDA. Poly(vinyl alcohol) (PVA, 87–90% hydrolyzed, $\bar{M_{W}\;}$ = 30,000–70,000 g mol^−1^) was used as an emulsifier-stabilizer; 1,1,1,3,3,3-hexafluoro-2-propanol (HFIP) by Fluorochem was used as solvent for viscometry measurements. All reagents were commercially available and were used as received.

### 2.2. Synthesis of PA 210 Microcapsules (PA MCs)

Polyamide microcapsules (PA MCs) were prepared via interfacial polymerization in an oil-in-water (O/W) emulsion, according to previous work [38]. The oil phase of the emulsion was prepared by mixing 1.8 mmol of SC in 1 mL of organic solvent (dodecane, hexane, toluene). This was added to 25 mL aqueous solution containing PVA 2% w/w and stirred vigorously at 1200 rpm using a mechanical stirrer (Axial 4 blade stirrer 5 cm diameter) for 3 min at room temperature. Then, 25 mL of an aqueous EDA (18 mmol) solution was added dropwise over 30 min at a stirring speed of 400 rpm. The diamine:dichloride (EDA:SC) molar ratio was equal to 10:1 in order to neutralize the formed byproduct (HCl). Furthermore, EDA is a highly volatile amine, so it was added in excess to compensate for potential loss during polycondensation. 

The final solution was left to react further for 30 min and then the PA MCs were collected through filtration and washed with deionized water to remove excess diamine and PVA. The final product was left to dry at room temperature in the fume hood and was received as colorless paste. The process yield of the interfacial polymerization was calculated by Equation (1):

(1) $$\mathrm{Process}\;\mathrm{yield}=\frac{m_{\exp}}{m_{\mathrm{theor}}}$$

where m_exp_ (g) is the experimental mass of the dried MCs, and m_theor_, the theoretical mass (g) calculated by polymerization mass balance.

A number of key process parameters, i.e., diamine:dichloride (EDA:SC) ratio, SC solvent and agitation speed, and time during polymerization were investigated herein (Table 1). A blank experiment (Control) without PVA was also conducted at a molar EDA:SC ratio of 10:1 (dodecane, 400 rpm, 30 min) for comparison reasons.

### 2.3. Synthesis of PA 210 Membranes (PA Membranes)

Polyamide membranes (ΡA membranes) were prepared via interfacial polymerization in order to examine the effect of the organic solvent (dodecane, hexane, toluene) in membrane robustness, mass yield and molecular weight. The organic solution comprised 2 mmol of SC dissolved in 2.5 mL of organic solvent, while the aqueous solution comprised of 20 mmol of EDA dissolved in 12.5 mL of deionized water. Τhe organic SC solution was slowly added to the aqueous EDA solution and left to react for 18 h [31]. The membrane was collected, washed with distilled water and dried at 60 °C in vacuum for 24 h. The conducted experiments differentiated only in the SC solvent used (dodecane, hexane, toluene); the resulted products are named respectively as M_D_, M_H_ and M_T_.

### 2.4. Hydrolysis of Sebacoyl Chloride under Emulsification Conditions

The hydrolysis of sebacoyl chloride during the emulsification step was examined; a solution of 50 mL PVA 2% w/w was combined with a solution of 2 mmol of SC in 2 mL dodecane and was stirred mechanically at 1200 rpm for 15 min. The pH was measured every minute with a pH electrode (HANNA Instruments). The degree of hydrolysis (%) was calculated by Equation (2):

(2) $$\mathrm{hydrolysis}\;\left(\%\right)=\frac{\left[H^{+}\right]}{2\times {\left[\mathrm{SC}\right]}_{0}}\;\times \;100$$

where [H^+^] the concentration of acid groups (mol L^−1^) calculated by pH values, and [SC]_0_ the initial concentration of SC (mol L^−1^).

### 2.5. Determination of Apparent Partition Coefficient of EDA

The partition coefficient of EDA in the SC solvents examined herein (dodecane, hexane, toluene) was measured in similar conditions with MC synthesis but without SC and PVA. Specifically, 1 mL of organic solvent was dispersed in 25 mL of water by stirring at 1200 rpm for 3 min. Then, 25 mL of an aqueous EDA (18 mmol) solution was added dropwise over 30 min at 400 rpm. At the end of this step, the emulsion was centrifuged for 10 min at 7500 rpm in order to separate the organic from the aqueous phase. Then, the aqueous phase was titrated with a HClO_4_ solution (N ~ 0.13 meq mL^−1^) in order to determine the amine content [NH_2_]_aq_ (meq kg^−1^). The final concentration of EDA (meq kg^−1^) in the aqueous phase is given by Equation (3).

[EDA]_aq_ = 0.5 × [NH_2_]_aq_ (3)

Then, the concentration of EDA in the organic phase [EDA]_org_ (meq mL^−1^) is calculated by Equation (4):

(4) $${\left[\mathrm{EDA}\right]}_{\mathrm{org}}=\frac{\left({\left[\mathrm{EDA}\right]}_{0\;}-{\left[\mathrm{EDA}\right]}_{\mathrm{aq}}\right)\times V_{\mathrm{aq}}}{V_{\mathrm{org}}}$$

where [EDA]_0_ (meq mL^−1^) is the initial concentration of EDA, V_aq_ (mL) the volume of the aqueous phase and V_org_ (mL) the volume of the organic phase.

Finally, the partition coefficient (*k*) is calculated as in Equation (5):

(5) $$k=\frac{{\left[\mathrm{EDA}\right]}_{\mathrm{aq}}}{{\left[\mathrm{EDA}\right]}_{\mathrm{org}}}$$

### 2.6. Characterization of PA 210 Microcapsules and Membranes

Microcapsule morphology was first examined by optical microscopy (Examet Union 82160, Union, Tokyo, Japan) at the end of the interfacial polymerization and prior to filtration. Digital images were recorded by Sony CCD-IRIS (SSC-C370P camera, Sony, Tokyo, Japan) and analyzed in ImageJ software in order to determine microcapsule size. To that end, at least 100 microcapsules were counted in order to determine the mean size and the polydispersity index (PDI) using Equation (6):

(6) $$\mathrm{PDI}=\frac{D_{\left(v,0.9\right)}-{\;D}_{\left(v,0.1\right)}}{D_{\left(v,0.5\right)}}$$

where, D_(v,0.5)_ is the particle size of which 50% of the sample is smaller and 50% is larger, D_(v,0.1)_ is the particle size of which 10% of the sample is smaller, D_(v,0.9)_ is the particle size of which 90% of the sample is smaller.

Scanning Electron Microscopy (SEM) was used to evaluate the most promising MC samples after filtration and drying using a Jeol 6300 JSM instrument which had a high sensitivity secondary electron detector and operated at 20 kV. The microcapsule powder was coated with Au coating using a Quorum Technologies SC7620 sputter coater with a sputtering time of 120 s at a current of 10 mA.

Fourier Transform Infrared Spectroscopy (FTIR) was used to determine the chemical structure of the microcapsules and membrane. Samples were mixed with KBr crystals and formed into pastilles. Measurements took place on a JASCO 4200 (JASCO, Gross-Umstadt, Hessen, Germany) instrument from 400 to 4000 cm^−1^ with a 4 cm^−1^ resolution.

The intrinsic viscosity ([*η*], dL g^−1^) of PA MCs and membranes was estimated by solution viscometry. Solutions of 0.1 g dL^−1^ in HFIP were measured in an Ubbelohde micro-viscometer at 25 °C (K = 0.0028 mm^2^ s^−2^). The [*η*] values were obtained by single point measurement, via Equation (7):

(7) $$\left[\eta \right]=\frac{\sqrt{1+1.5\eta_{sp\;}}+1}{0.75C}$$

where *η*_sp_ is the specific viscosity and C is the solution concentration (g dL^−1^). 

An estimation of the weight-average molecular weight ($\bar{M_{w}}$, g mol^−1^) was calculated using the Mark-Houwink-Sakurada equation (Equation (8)):

(8) $$\left[\eta \right]=K{\bar{M_{w}}}^{a}$$

assuming that K and a, the Mark Houwink parameters, are close to the relevant values for PA 66 solutions in HFIP at 25 °C (K = 0.00198 and a = 0.64) [47].

The thermal stability of the capsules was assessed by thermogravimetric analysis (TGA) in a Mettler Toledo TGA/DSC 1 thermobalance. The microcapsules were heated from 30 to 550 °C under nitrogen flow (25 mL min^−1^) at a heating rate of 10 °C min^−1^. The onset decomposition temperature was defined as the temperature at 5% mass loss (*T*_d5%_). Degradation temperature (*T*_d_) was obtained for the maximum mass loss rate and the char yield as the % residue at 550 °C.

Differential Calorimetry (DSC) runs also took place in a Mettler DSC 1 Star^e^ System. Samples were inserted at 30 °C, preheated at 100 °C, then cooled at 25 °C. From there, a heating-cooling-heating cycle from 25 °C to 300 °C with a heating rate of 10 °C min^−1^ was employed. The melting point (*T*_m_) and the specific heat of fusion (Δ*H*_m_) were obtained by data from the second heating, while the melt crystallization temperature (*Τ*_c_) and enthalpy (Δ*H*_c_) were obtained from the cooling step.

## 3. Results and Discussion

### 3.1. Selection of Interfacial Polymerization Critical Conditions—MC1

Our first efforts were to determine the experimental conditions which permit the formation of robust polyamide microcapsules via interfacial polymerization while achieving a satisfactory process yield. MC1 microcapsules were prepared with dodecane as solvent for SC, which also acts as core material. PVA was selected as stabilizer according to previous work, and the stirring speed during the emulsification step was kept constant at 1200 rpm in order to achieve particle size below 150 μm [38].

**Figure 1:**
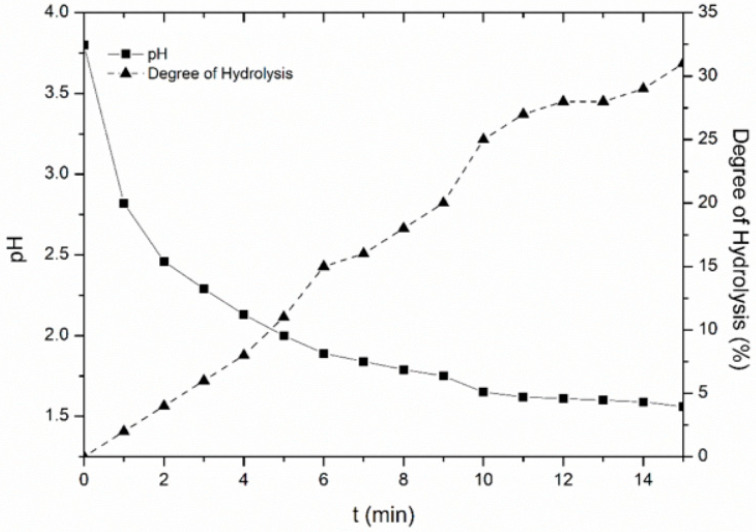
pH and degree of hydrolysis of sebacoyl chloride under emulsification conditions.

Interfacial polymerization consists of two main steps: the emulsification step, i.e., the dispersion of the organic phase containing the dichloride in the stabilizer aqueous solution (PVA 2% w/w), then the polymerization step, starting with the addition of the diamine. The yield of the process is mainly controlled by the second step. However, during the emulsification hydrolysis of acyl chloride occurs as a side reaction, which limits the amount of chloride groups left to react, thereby lowering the reaction yield [29,31,38]. This is valid for the case of SC despite the fact that it is considered one of the less sensitive acyl chlorides to hydrolysis [6,38]. In this context, a study on the SC hydrolysis was conducted herein to determine the appropriate emulsification time: low emulsification time restricts acyl chloride hydrolysis; however, long emulsification times lead to microcapsules with smaller size and more narrow size distribution [25]. As anticipated, the hydrolysis of SC herein generated hydrochloric acid which, in absence of a base, led to a decrease in pH (Figure 1). The percentage of SC hydrolysed was stoichiometrically calculated (Equation (2)) and the degree of hydrolysis was found to increase almost linearly with time. This linear behavior is similar to a previous study of SC hydrolysis where it was found that, after 8 min of emulsification, SC was almost fully hydrolyzed [38]. On the contrary, in our case the hydrolysis rate was found to be much slower as less than 20% of SC was hydrolyzed after 8 min; such deviation may be attributed to the different experimental conditions including the type and the geometry of the stirrer, and the dimensions of the reaction vessel [7,26]. In order to compromise between time required for proper emulsification and SC hydrolysis an emulsification time of 3 min was chosen for our experimental procedure, as only 6% of SC was hydrolyzed.

**Figure 2:**
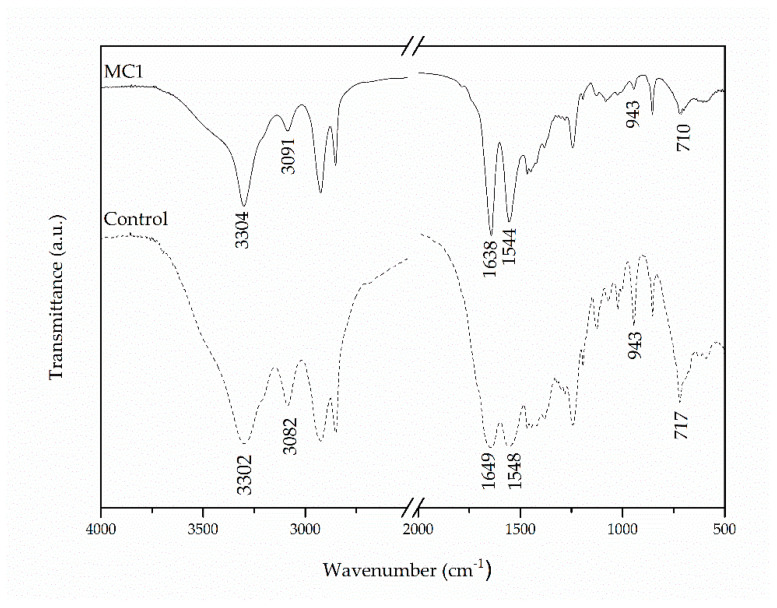
FTIR spectra of MC1 and Control samples.

Starting with polyamide MC preparation, the process of interfacial polymerization was firstly examined in terms of synthesized PA 210 characteristics, i.e., chemical structure, process yield, molecular weight and thermal properties. Based on FTIR, the MC1 spectrum confirmed polyamide formation (Figure 2). More specifically, when compared to the Control sample both spectra included the characteristic peaks for polyamides: at 3302 and 3304 cm^−1^ (hydrogen-bonded-NH stretching vibration-Amide Band I), at 3082 and 3091 cm^−1^ (Amide B overtone of Amide II), at 1649 and 1638 cm^−1^ (–C–CO stretching vibration-Amide Band I), at 1548 and1544 cm^−1^ (–CN stretching vibration and –CONH bend-Amide Band II), at 943 cm^−1^ (–C = O stretching vibration-Amide Band IV), and at 717 and 710 cm^−1^ (–NH out of plane bend). In addition, the absence of the vibrational band corresponding to the group –COCl from sebacoyl chloride (approx. at 1800 cm^−1^) suggests that SC was consumed. Polyamide formation was confirmed for all further MCs investigated in this study, with negligible shifts in the positioning of peaks (Supplementary Figure S1).

With respect to polymerization efficiency, a low process yield was obtained (11%) for MC1, significantly reduced compared to the Control experiment (42%) (Table 2). Apart from SC hydrolysis, this low yield can be correlated to the ability of EDA to dissolve into organic solvent droplets through the polyamide membrane. It is accepted that interfacial polymerization is a diffusion-controlled process and the rate-controlling step is EDA diffusion into the organic phase under polymerization conditions [30,31,36,39,48]. EDA is a highly hydrophilic monomer with great affinity for the aqueous phase [36], which could account for the low yield. To quantify this effect, the partition coefficient (*k*) of EDA in dodecane was experimentally determined for the case of MC1: *k* was found equal to 2.0, which is considerably lower than the one calculated by Zydowicz et al. for EDA in 3:1 cyclohexane/chloroform mixture, which was equal to 31.2 [36]. It should be stated, though, that the tendency of EDA to dissolve into the organic phase is influenced by the nature of the organic solvent, and the partition coefficient is highly dependent on the experimental conditions applied [31,48]. Indicatively, our value is close to the one calculated in the work of Soares Latour et al. for tetramethylene diamine in jojoba oil, which was 1.2 [31].

The low process yield of MC1 was accompanied by a low attained molecular weight ($\bar{M_{w}}$ = 3600 g mol^−1^) as evidenced by the measured intrinsic viscosity. MC1 was an oligoamide presenting lower [*η*] (0.334 dL g^−1^) compared to the Control sample (0.550 dL g^−1^). The molecular weight of the polyamide microcapsules is not often quoted in literature, as the use of polychlorides and polyamines leads to branched polyamide shells that cannot be dissolved in common viscometry or chromatography solvents. However, for cases of aliphatic polyamide microcapsules, it has been reported that the shell is in fact composed of oligoamides [31].

**Figure 3:**
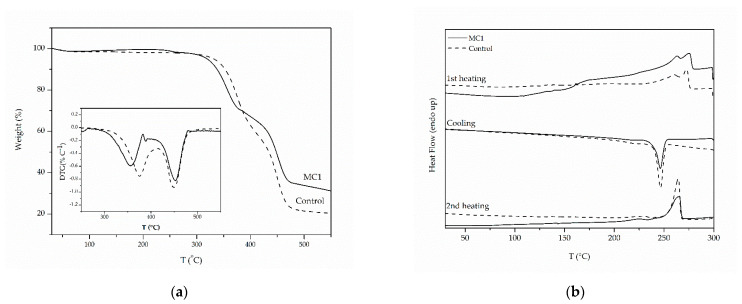
TGA (**a**) and DSC (**b**) curves of MC1 and Control samples after filtration and drying.

Regarding MC1 thermal properties, no mass loss was observed in the TGA graph (Figure 3a) at the dodecane boiling point (*T*_boiling_= 216 °C), indicating that the microcapsules had collapsed and the majority of the core liquid (dodecane) had already escaped. A two-step degradation profile was noted: the first step occurred at 365 °C and the second at 453 °C, while the same profile was also found for the Control sample at higher respective temperatures (375 °C and 449 °C) (Table 3). This finding of two-step degradation is not typical for polyamides, whose degradation usually occurs in a single step. However, studies in polyamides with short chain length diamines (PA 313, PA 320, PA 218) have revealed that decomposition starts at relatively low temperatures (350 °C) due to their low molecular weights ([*η*] < 0.43 dL g^−1^) and the instability of the amines, e.g., in the case of PA 320 two degradation steps have been observed, at 360 °C and at 433 °C [49,50]. Relatively high residue values were observed for both MC1 and Control samples (20% and 31%, respectively), which was also the case for some polyamides with short aliphatic amine [50]. This could be attributed to the instability of the amines and cyclization reactions.

On the DSC curves of MC1, during the first heating a broad endotherm is observed at 150–180 °C (Figure 3b). This could be attributed to the evaporation of dodecane traces from the sample. Double melting peaks are apparent at 262 °C and 275 °C for MC1 and at 261 °C and 273 °C for the Control experiment (Table 3). As previously reported, polyamides 2Y combine both a- and β- crystalline structures; thus, this behavior could be associated with the melting of different crystalline forms [49,51]. The data from the cooling and the second heating cycle refer to the pure PA 210 shell. During the cooling cycle a single sharp melt crystallization peak appeared at 246 °C for MC1 and at 245 °C for the Control sample, indicating similar molecular size for both samples. Similarly, the melting points of MC1 and Control experiment at the second heating cycle were close (265°C and 264 °C respectively), proving again that both products are of similar molecular weight, as also shown by [*η*] values.

**Figure 4:**
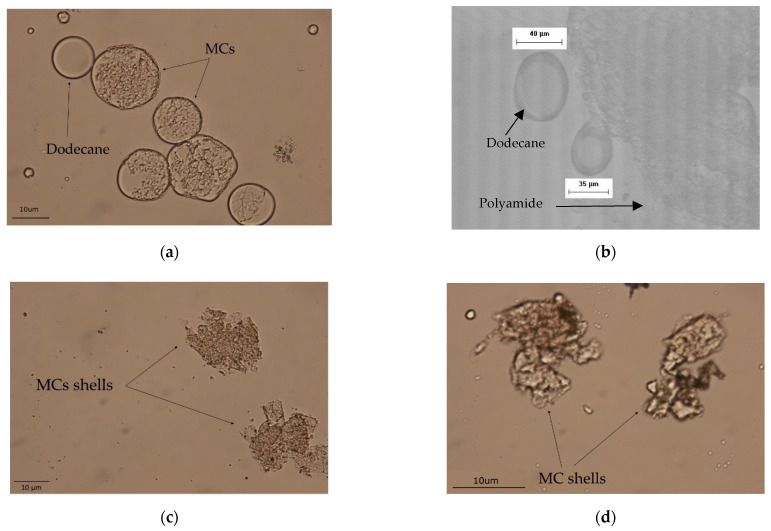
Morphology of (**a**) MC1 prior to filtration, (**b**) Control experiment after filtration (**c**) MC1 after filtration and (**d**) MC1 after drying at 40 °C and redispersion.

**Figure 5:**
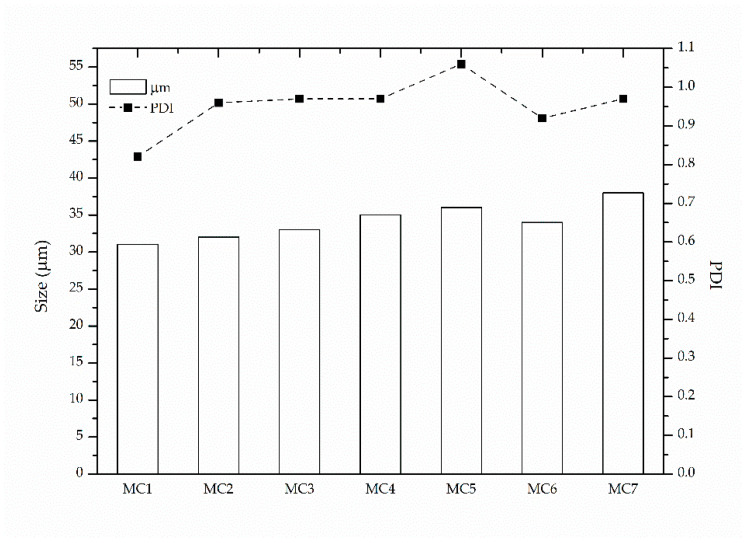
Mean size and calculated polydispersity index of MCs based on optical microscopy data and using ImageJ software.

Turning to the morphology of MC1, prior to filtration, dark spherical structures were observed via optical microscopy, confirming the formation of microcapsules (Figure 4a). The size of MC1 was assessed via ImageJ software at a range 9–75 μm with an average value of 33 μm, which is considerably lower than the reported value in the literature (153 μm), probably due to the different type of stirrer and experimental apparatus used therein [27,38]. The relatively high PDI value (PDI = 0.82) confirmed that the sample was polydisperse (Figure 5) [52]. On the other hand, the Control experiment yielded solid PA 210 in the form of flakes, which appeared as a shapeless dark mass in optical microscopy (Figure 4b) after washing and filtration. However, MC1 microcapsules presented numerous fractures and incomplete shells with many droplets of dodecane not encapsulated. This was further observed as MC1 microcapsules were unable to maintain their morphology during filtration (Figure 4c), proving their brittle nature. This collapse was enhanced after drying (40 °C) (Figure 4d), something that hindered MC redispersion in water for optical microscopy of the received product.

**Figure 6:**
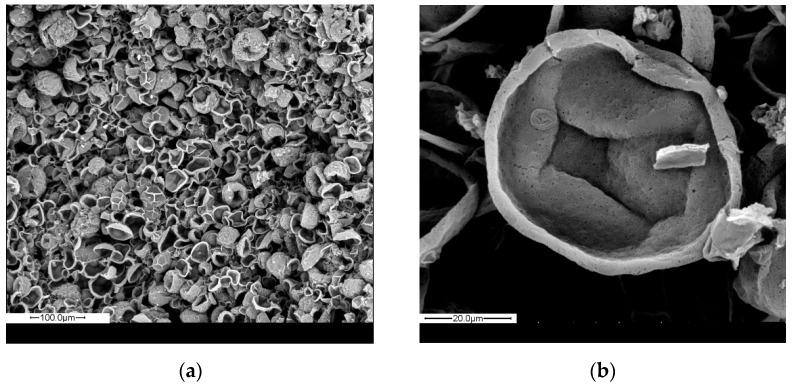
SEM images of MC1 after washing with water, filtration and drying at 40 °C (**a**) magnification 200× (**b**) magnification 1600×.

SEM images of MC1 after filtration supported the above discussion, as collapsed spherical shells were observed (Figure 6a). The average size was estimated via ImageJ software from 100 microcapsules and was found at 31 μm, close to the one calculated from optical microscopy. The thickness of the polyamide shell was also calculated similarly and was found at 3.1 ± 0.5 μm (Figure 6b).

Shell fragility is common in polyamide microcapsules and can be attributed to a number of factors, including limited amine diffusion through the organic phase, low molecular weight and very high shear rate during the polymerization step [24,27]. As this is a major obstacle when considering the potential application of polyamide microcapsules, the following part of this work was to overcome the existing problem of shell integrity prior to MC filtration by increasing the achieved molecular weight. Critical process parameters were examined, such as the EDA:SC molar ratio (samples MC1, MC2), the effect of SC organic solvent (samples MC1, MC3 and MC4), and the stirring speed during the polymerization step (samples MC1, MC5, MC6). The appropriate conditions were combined to prepare MC7 (Table 1).

### 3.2. Effect of Monomers Molar Ratio on Polyamide Microcapsules—MC2

The molar ratio of the monomers (amine:chloride) has been found to significantly influence the formation of the polyamide membrane in typical interfacial polymerization processes [6,36,39]; this ratio is not set equal to 1 as the system is heterogenous and controlled by the diffusion of the water-soluble monomer (EDA in our case) through the membrane. Therefore, an excess of this monomer is tolerated in order for diamine to be continuously able to diffuse through the membrane and create the necessary molar equilibrium in the reaction zone [6]. Furthermore, in order to achieve high yields it is important to neutralize the reaction byproduct. In other words, the excess of amine used herein also serves to neutralize the hydrochloric acid produced during the polycondensation reaction. Generally, an amine:chloride ratio equal to 5:1 is applied [27,31,39,48]. However, the excess of strong acid acceptors has been found to negatively influence the yield as well as the viscosity of the produced polyamides [36,48]. Therefore, we reduced the ratio to 5:1 (MC2) from 10:1 (MC1).

However, in our PA 210 MCs, lowering the amine:chloride ratio did not have the desired effect as the process yield decreased significantly to 3%, compared to 11% in MC1 (Table 2). Apparently, the amine content was not enough to act both as a neutralizing agent for the byproduct and as a monomer for the reaction. Furthermore, EDA is particularly volatile, so a part of it could have also evaporated during the course of the reaction. The formation of the polyamide shell was confirmed by FTIR (Figure S1b); however, a 32% decrease in the molecular weight of MC2 was also observed, further proving that there was not enough amine for reaction. The thermal properties of MC2 revealed earlier decomposition, as the *T*_d,5%_ decreased from 319 °C to 317 °C and the *T*_d1_ decreased from 365 °C to 362 °C (Figure S2b), probably due to the lower molecular weight of the product. No significant changes in the melting or crystallization temperatures were observed (Table 3).

**Figure 7:**
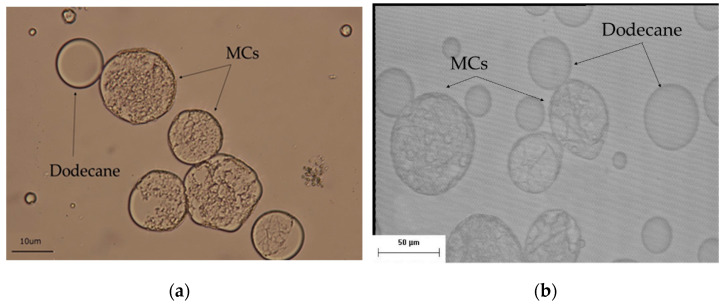

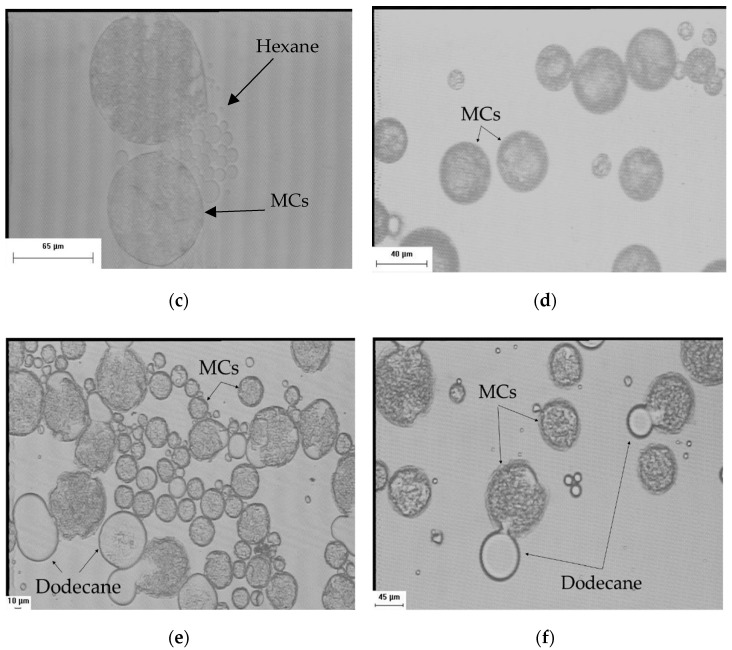
Morphology of (**a**) MC1; (**b**) MC2 (ratio 5:1); (**c**) MC3 (hexane) (**d**) MC4 (toluene); (**e**) MC5 (100 rpm, 0.5 h); (**f**) MC6 (100 rpm, 2 h). All images refer to MCs prior to filtration.

As to the morphology of MC2, the optical microscopy of the emulsion (prior to filtration) revealed polyamide shells which cracked immediately, releasing the encapsulated dodecane (Figure 7b). The size of the produced MCs remained practically unchanged at 32 μm with a PDI value of 0.95 (Figure 5). This was expected, as the size and size distribution of MCs are mainly influenced by the type and concentration of the emulsifier and the agitation speed during the emulsification step, which were kept constant compared to MC1. It is therefore evident that it is necessary to keep the amine:chloride ratio equal to 10 in order to achieve an acceptable yield and molecular weight.

### 3.3. Effect of SC Solvent/Organic Core on Polyamide Microcapsules—MC1, MC3, MC4

The precipitation and the formation of the membrane of microcapsules is affected by the type of the organic solvent used for the dissolution of the acyl chloride. Polymerization rate is controlled by the mass transfer of the diamine, as the growth of the membrane occurs towards the organic phase in the case of oil-in-water emulsions [2,6,36]. The interactions between polymer and solvent influence not only the morphology and the thickness of the polymeric shell, but also the molecular weight of the polymer. Consequently, the selection of the organic solvent deserves special attention. For that purpose, three different experiments were conducted herein, each of them with a different solvent: dodecane (MC1), hexane (MC3), toluene (MC4).

The process yield deteriorated significantly in the case of hexane (MC3); it was decreased to 1% from 11% (MC1, dodecane) (Table 2). However, switching to toluene (MC4) almost tripled the process yield, from 11% to 30%. The calculation of the partition coefficient (*k*) of EDA in dodecane (MC1), hexane (MC3) and toluene (MC4) confirmed the aforementioned data, as for MC1 *k* was found equal to 2.0, for MC3 it was 2.9 and for MC4 it was 1.7, meaning in each case that the diffusion of EDA was harder for MC3, less hard for MC1 and easiest for MC4. It is obvious at this point that increasing the polarity of the solvent increases the process yield, which is in agreement with the fact that higher-polarity organic solvents such as toluene are preferred due to their presenting higher partition coefficients and facilitation of membrane precipitation, in comparison to lower polarity solvents [6,48,52]. Another explanation for the higher yield in the case of MC4 may be correlated with the solubility of the polyamide in the solvent. It has been reported that in interfacial polymerization reactions the oligomers produced in the early stages should have a degree of solubility in the organic phase in order to allow the polycondensation to proceed in the organic phase [2,6,48,52,53,54]. However, the solvents should also aid the precipitation of the polyamide to an appropriate degree of polymerization in order to form the polymeric shell. Considering that the solubility parameters (***δ***) for the used solvents are: Hexane (14.9 MPa^1/2^), Dodecane (16 MPa^1/2^), Toluene (18.3 MPa^1/2^), and that for a typical polyamide such as PA 66 ***δ*** is about 28 MPa^1/2^, it is noted that the highest yield is observed for the toluene, whose δ is the closer to that value. The results for the molecular weight measurements followed the same trend; however, they were less conclusive. The molecular weight of MC3 was 45% lower than MC1, while the molecular weight of MC4 was only 4% higher than MC1 (Table 2). 

**Figure 8:**
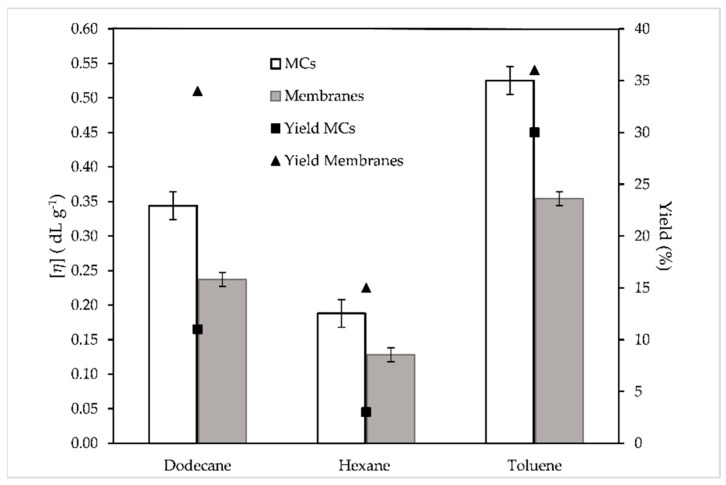
Intrinsic viscosity and process yield values of MCs and membranes produced by three different organic solvents.

The effect of organic solvent was also investigated by model membranes and similar trends were observed (Figure 8). The highest values both for the yield and for the intrinsic viscosity were observed for the membrane produced with toluene as the organic solvent M_T_ (36% and 0.354 dL g^−1^, respectively), followed by the one produced with dodecane (M_D_) and lastly the one produced with hexane (M_H_). However, it must be noted that while their yields are significantly larger, the produced membranes tend to have lower [*η*] than the microcapsules (Figure 8). This might be attributed to the larger surface area between the organic and the aqueous phase available in the case of MCs due to the formation of a large number of organic droplets. The synthesis of model membranes is indeed a useful tool for estimating qualitatively the properties of the microcapsules; however, the quantitative image is not so conclusive and precise.

**Figure 9:**
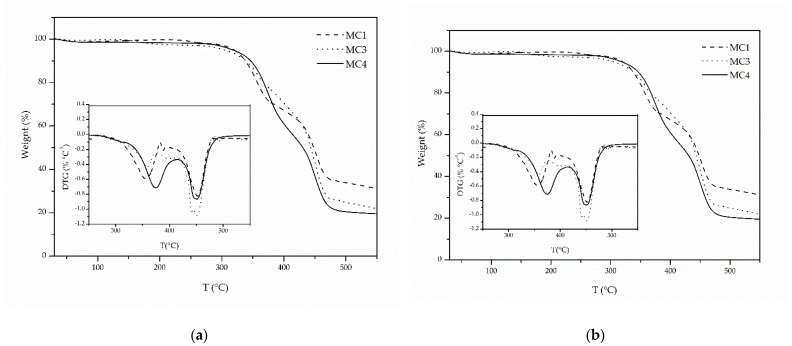
TGA graphs of (**a**) MC1, MC3 and MC4 and (**b**) their respective membranes M_D_, M_H_, M_T_. All samples are MCs and membranes respectively after filtration and drying.

It is also worth noting that the degradation profiles of MC1, MC3 and MC4 match those of their respective membranes (Figure 9). In both MC3 and M_H_ degradation started earlier (*T*_d5%_ equal to 305 °C for MC3 and 276 °C for M_H_), and in MC4 and M_T_ it started later (*T*_d5%_ 318 °C for MC4 and 312 °C for M_T_), as is also indicated by their TGA curves (Table 3, Figure 9b). This further proves the validity of membrane formation as a tool for qualitatively estimating polyamide microcapsule properties. It must be stated, though, that in the TGA curves of MCs, no step associated with core evaporation was observed in the range of solvent boiling points; therefore, it is evident that the microcapsules have cracked upon filtration and the majority of the core was again released.

Turning to the morphological aspects of the MCs, MC4 produced more complete and rigid polyamide capsules, as indicated by the dark spherical shapes in optical microscopy (Figure 7d). Furthermore, fewer dodecane droplets were observed, proving that the overall structure was more robust and that the core was encapsulated to a higher degree. The exact opposite was observed for MC3 (Figure 7c), as the polyamide shell was very fragile and numerous droplets of hexane were observed. The mean size of the MCs was 33 μm for MC3 and 35 μm for MC4, with a PDI of 0.97 in each case (Figure 5). Therefore, it can be concluded that toluene is the most suitable solvent for encapsulation as it yields microcapsules with higher yield and molecular weight as well as better morphology prior to filtration compared to dodecane and hexane.

### 3.4. Effect of Polymerization Stirring Rate and Time on Polyamide Microcapsules—MC5, MC6

As in the previous experiments, the capsules produced seemed to have various cracks and released the encapsulated solvent; the effect of stirring speed during the polymerization step was examined as a parameter affecting shell integrity. Higher stirring speed is associated with high shear rate, which might cause the microcapsules to crack and collapse [6,24]. Therefore, lowering the stirring speed from 400 rpm (ΜC1) to 100 rpm (MC5) should prevent this while maintaining MC size, as this is determined by the emulsification step.

As to the process yield, lowering the stirring speed had little effect and the yield increased slightly, from 11% to 14% (Table 2). This was to be expected, since the diffusion of EDA, which is the rate-determining step, is primarily influenced by the nature and ratio of the monomers as well as the organic solvent. MC5 and MC1 presented very similar molecular weights (0.313 dL g^−1^ and 0.344 dL g^−1^), indicating few differences between the two grades (Table 2). Turning to the thermal properties, the melting and crystallization temperatures of MC5 were found slightly lower than those of MC1 (262 °C compared to 265° C and 242 °C compared to 246 °C) (Table 3). TGA results also confirmed similar degradation temperatures for MC5 and MC1 (*T*_d5%_ at 318 °C and 319 °C and *T*_d1_ at 367 °C and 365 °C, respectively). 

Optical microscopy of the emulsions, however, proved the effectiveness of decreasing the stirring speed in improving the microcapsule morphology (Figure 7e). MC5 shows the formation of complete opaque microcapsule shells with only a few droplets of non-encapsulated dodecane. The size and size distribution of the microcapsules remains practically unchanged (36 μm) with a PDI of 1.06 (Figure 5). This small increase in PDI is, however, within error deviations. This stirring speed was kept throughout the following experiments.

Along with lowering the stirring speed, the effect of polymerization time was also studied (MC6), by increasing the polymerization time from 30 min (MC5) to 2 h (MC6). Reaction rate is dependent on the duration of the polymerization step and longer polymerization times tend to yield thicker membranes; however, the length of reaction time varies with the type of monomer [42].

The yield of the process remained unaffected at 11%. This was also the case reported by Soto-Portas et al. when examining the effect of reaction time on the synthesis of Jeffamine based MCs [28,39]. However, a 16% increase in molecular weight was noted (from 0.344 dL g^−1^ to 0.398 dL g^−1^) due to the longer time allowed for the diffusion and reaction of EDA (Table 2). This was accompanied by a 9% increase in the cold crystallization enthalpy (from 56 J g^−1^ to 61 J g^−1^) and in the *T*_d,5%_ (from 319 °C to 324 °C) (Table 3). The morphology of MC6 was found similar to MC5, as opaque spherical microcapsule structures were observed (Figure 7f) and their mean size was at 34 μm with a PDI of 0.92. However, non-encapsulated dodecane droplets were observed, along with cracked shells more prominent than in MC5, leading us to believe that long exposure even at this shear rate might damage the microcapsule shell.

### 3.5. Implementation of Most Appropriate Conditions for PA 210 MCs Formation via Interfacial Polymerization—MC7

In an effort to combine the most appropriate process parameters, MC7 was conducted by implementing the aforementioned conclusions regarding interfacial polymerization conditions, i.e., amine:chloride ratio equal to 10, use of toluene as the organic solvent and setting the stirring speed at 100 rpm and time at 30 min during the polymerization step.

The process yield in MC7 was found close to the one observed for MC4, and was the highest overall at 33%. Along with the highest yield, the highest molecular weight was also observed for MC7 at 0.525 dL g^−1^ (Table 2), achieving an overall 52% increase compared to MC1. As to MC7 thermal properties, the crystallization temperature was raised from 246 °C to 251 °C compared to MC1, along with the crystallization enthalpy which was raised from 56 J g^−1^ to 88 J g^−1^ (Table 3). Although no significant changes were recorded in the melting points, the melt enthalpy was increased from 57 J g^−1^ to 79 J g^−1^. Finally, *T*_d1_ increased from 365 °C to 374 °C, proving the overall improvement of thermal properties, which is linked to the increase of the molecular weight.

**Figure 10:**
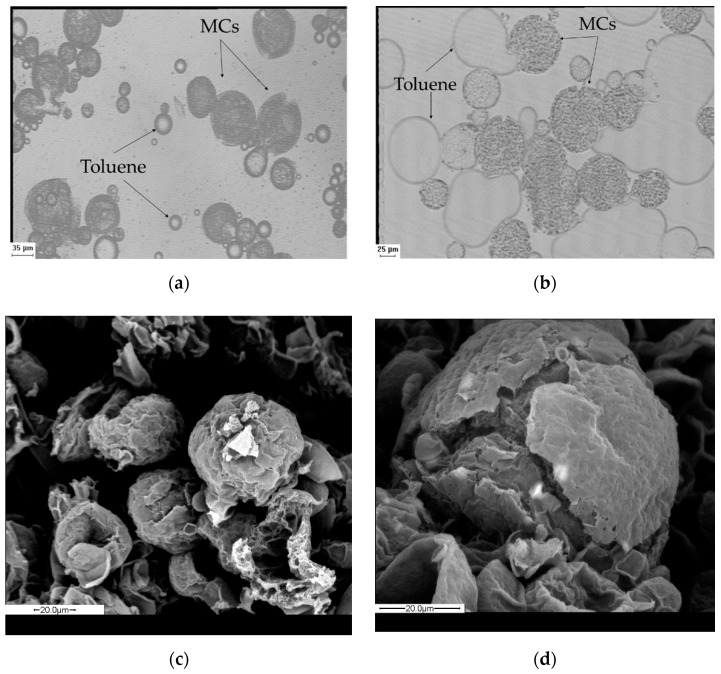
Morphology of (**a**) MC7 prior to filtration, (**b**) after filtration and redispersion (**c**,**d**) SEM images of MC7 after filtration.

Optical microscopy of MC7 prior to filtration revealed the formation of complete polyamide microcapsule shells (Figure 10a) with a size between 15–90 μm and a mean value of 38 μm and a PDI of 0.97. After filtration the microcapsules still cracked and released the encapsulated toluene (Figure 10b). However, there was some improvement from the case of MC1 (Figure 4c), where only polyamide shells were visible. This was also supported by SEM images of MC7, as again mostly collapsed polyamide shells were observed (Figure 10c,d). This might also be partly attributed to the high volatility of toluene, which could evaporate during the preparation of SEM samples. Although we succeeded in increasing PA 210 shell stability, therefore permitting the use of MCs as an aqueous dispersion directly after the interfacial polymerization, isolation of dried MCs remains a challenge.

## 4. Conclusions

In the present work, the synthesis of aliphatic polyamide (PA 210) microcapsules via interfacial polymerization was examined. Taking into consideration the fragility of the microcapsules, the effects of key process parameters were examined, such as the ratio of monomers, the core solvent, and the stirring speed and time in the polymerization step. As a first step, the appropriate emulsification time was determined based on the kinetics of sebacoyl chloride hydrolysis. The formation of PA 210 MCs in dodecane resulted in a low process yield (11%) and MCs presented incomplete shell formation directly after interfacial polymerization. Lowering the amine:chloride ratio from 10:1 to 5:1 was not found efficient in terms of yield, molecular weight or MC morphology, as the available amine was insufficient for reaction and neutralization of the reaction byproduct. The nature of the organic core plays an important role; switching from dodecane to toluene almost tripled the process yield and also led to a small increase in molecular weight. The results were correlated with the diffusion of EDA in the organic solvent by calculating the relevant partition coefficients. The effect of core solvent was also studied by model (i.e., plane) membranes and both the yield and the molecular weight results followed the same trend as the corresponding MCs experiments. Lowering the stirring from 400 rpm to 100 rpm did not affect the yield, molecular weight or the thermal properties of the MCs; on the other hand, a less defective MC morphology was attained. An increased polymerization time did not affect yield and only led to a small increase in molecular weight. Present optimum results correspond to MCs with a yield of 33% and a PA 210 molecular weight of 7000 g mol^−1^; MCs retain their morphology in aqueous dispersion, while isolation of good quality dried MCs remains a challenge.

## Figures and Tables

**Table 1:** Experimental parameters for PA 210 microcapsules (PA MCs) synthesis via interfacial polymerization.

Sample	EDA:SC Molar Ratio	SC Solvent	Agitation Speed (rpm)	Agitation Time (min)
MC1	10:1	Dodecane	400	30
MC2	5:1	Dodecane	400	30
MC3	10:1	Hexane	400	30
MC4	10:1	Toluene	400	30
MC5	10:1	Dodecane	100	30
MC6	10:1	Dodecane	100	120
MC7	10:1	Toluene	100	30

**Table 2:** Process yield, intrinsic viscosity ([*η*]), weight-average molecular weight ($\bar{M_{w}}$) of PA 210 MCs.

Sample	Process Yield (%)	[*η*] (dL g^−1^)	$\bar{M_{w}}\;(g\;{\mathrm{mol}}^{-1})$
Control	42	0.550	7500
MC1	11	0.344	3600
MC2	3	0.233	2000
MC3	1	0.188	1400
MC4	30	0.372	4100
MC5	14	0.313	3100
MC6	11	0.398	4500
MC7	33	0.525	7000

**Table 3:** Thermal properties (DSC and TGA) of PA 210 MCs.

DSC					TGA			
Sample	*T*_c_ (°C)	Δ*H*_c_ (°C)	*T*_m_ (°C)	Δ*H*_m_ (J g^−1^)	*T*_d5%_ (°C)	*T*_d1_ (°C)	*T*_d2_ (°C)	Residue (%)
Control	245	69	264	83	332	375	449	20
MC1	246	56	265	57	319	365	453	31
MC2	245	56	266	59	317	362	450	27
MC3	244	66	264	65	304	358	451	22
MC4	251	60	266	57	318	375	450	20
MC5	242	55	262	60	318	367	454	28
MC6	243	61	263	59	324	360	451	38
MC7	251	88	266	79	305	374	452	22

## Data Availability

Data is contained within the article and Supplementary Material.

## Supplementary Materials

The following are available online at www.mdpi.com/article/10.3390/ma14195895/s1, Figure S1: FTIR graphs of MCs after filtration and drying: (a) effect of monomers molar ratio, (b) effect of SC solvent/ organic core, (c) effect of polymerization stirring rate and time and (d) implementation of most appropriate conditions; Figure S2: TGA curves of MCs after filtration and drying: (a) effect of monomers molar ratio, (b) effect of SC solvent/ organic core, (c) effect of polymerization stirring rate and time and (d) implementation of most appropriate conditions.
